# Beyond IBD: the genetics of other early-onset diarrhoeal disorders

**DOI:** 10.1007/s00439-023-02524-6

**Published:** 2023-02-14

**Authors:** Lorraine Stallard, Iram Siddiqui, Aleixo Muise

**Affiliations:** 1 SickKids Inflammatory Bowel Disease Centre, The Hospital for Sick Children, Toronto, ON Canada; 2 Division of Pathology, The Hospital for Sick Children, Toronto, ON Canada; 3 Cell Biology Program, Research Institute, The Hospital for Sick Children, Toronto, ON Canada; 4 Department of Pediatrics, Institute of Medical Science and Biochemistry, University of Toronto, The Hospital for Sick Children, Toronto, ON Canada

## Abstract

Diarrhoeal disorders in childhood extend beyond the inflammatory bowel diseases. Persistent and severe forms of diarrhoea can occur from birth and are associated with significant morbidity and mortality. These disorders can affect not only the gastrointestinal tract but frequently have extraintestinal manifestations, immunodeficiencies and endocrinopathies. Genomic analysis has advanced our understanding of these conditions and has revealed precision-based treatment options such as potentially curative haematopoietic stem cell transplant. Although many new mutations have been discovered, there is frequently no clear genotype–phenotype correlation. The functional effects of gene mutations can be studied in model systems such as patient-derived organoids. This allows us to further characterise these disorders and advance our understanding of the pathophysiology of the intestinal mucosa. In this review, we will provide an up to date overview of genes involved in diarrhoeal disorders of early onset, particularly focussing on the more recently described gene defects associated with protein loosing enteropathy.

## Introduction

Diarrhoea in childhood is common and usually self-limiting. However, there are persistent and severe forms of diarrhoea due to monogenic disorders that frequently present within the first weeks of life. The term *congenital diarrhoea and enteropathies* (CODEs) is used to describe these often life-limiting forms of diarrhoeal disorder. In recent years, we have developed a greater understanding of the underlying genetic basis and pathophysiology of these disorders due to the availability of next generation sequencing. Fundamentally most identified gene defects involve intestinal epithelial and/or immune function and can be classified based on a common biological category. Although there is considerable overlap between categories, they can be grouped into epithelial barrier and response defects, epithelial enzymes and metabolism defects, T and B cell differentiation defects, hyper and auto-inflammatory disorders and regulatory T cells and immune regulation (Table 1). Monogenic disorders are thought to account for the majority of CODEs with sustained symptoms (Thiagarajah et al. 2018). Additionally, in children with very early-onset inflammatory bowel disease (VEO-IBD), studies have shown monogenic defects in 7.8% (Crowley et al. 2020) and in 32% of those with severe disease (Charbit-Henrion et al. 2018). Hence, genetic testing should be employed early in the diagnostic work-up to improve patient care and provide better prognostication.

**Table 1:** Classification of monogenic diarrhoeal disorders

Biological category	Chrom	Gene	Inheritance	OMIM no.
Epithelial barrier and response defects				
TTC7A deficiency	chr2	TTC7A	AR	609332
ALPI deficiency	chr2	ALPI	AR	171740
PLVAP deficiency	chr19	PLVAP	AR	607647
Microvillus inclusion disease	chr18	MYO5B	AR	606540
Tufting enteropathy	chr2	EPCAM	AR	185535
Congenital sodium diarrhoea	chr12	GUCY2C	AD	601330
Congenital sodium diarrhoea	chr5	SLC9A3	AR	616868
Congenital chloride diarrhoea	chr7	SLC26A3	AR	126650
AIFBL2	chrX	ELF4	XL	300775
Epithelial enzymes and metabolism				
DGAT1 deficiency	chr8	DGAT1	AR	604900
Congenital lactase deficiency	chr2	LCT	AR	603202
Sucrase-isomaltase deficiency	chr3	SI	AR	609845
Abetalipoproteinaemia	chr4	MTTP	AR	157147
Chylomicron retention disease	chr5	SAR1B	AR	607690
T and B cell differentiation defects				
ARPC1B deficiency	chr7	ARPC1B	AR	604223
DOCK8 deficiency	chr9	DOCK8	AR	611432
LRBA deficiency	chr4	LRBA	AR	606453
STAT1 GOF	chr2	STAT1	AD	600555
STAT3 GOF	ch17	STAT3	AD	102582
SYK GOF	chr9	SYK	AD	600085
Hyper and auto-inflammatory disorders				
XIAP	chrX	XIAP	XL	300079
CD55 deficiency	chr1	CD55	AR	125240
CGDX	chrX	CYBB	XL	300481
HPS1	chr10	HPS1	AR	604982
Regulatory T cells and immune regulation				
IPEX	chrX	FOXP3	XL	304790
IL10/IL10R deficiency	chr21	IL10RB	AR	123889
CTLA4 haploinsufficiency	chr2	CTLA4	AD	123890
APECED	ch21	AIRE	AR	607358

*AD* autosomal dominant, *AR* autosomal recessive, *XL* X-linked, *OMIM* Online Mendelian Inheritance in Man, *AIFBL1* auto-inflammatory syndrome, familial, X-linked, Behcet-like 2, *GOF* gain of function, *CGDX* chronic granulomatous disease x-linked, *HPS1* Hermansky–Pudlak syndrome 1, *IPEX* immune dysregulation, polyendocrinopathy, enteropathy, X-linked, *APECED* autoimmune polyendocrinopathy-candidiasis-ectodermal dystrophy

Here, we describe a number of genes from each of these broad categories and particularly the more recently described gene defects associated with protein-losing enteropathy.

## Protein-loosing enteropathy

Protein-losing enteropathy (PLE) is a diarrhoeal disorder characterised by excessive protein loss through the GI tract, due to the disruption of the intestinal mucosal membrane or dilatation of the intestinal lymphatic system (Braamskamp et al. 2010). Patients typically present with hypoalbuminaemia and oedema. Albumin loss into the intestine may reach up to 60% of the total albumin pool (normal 15%) (Lee and Sung 2004). PLE has varied consequences including fat malabsorption, fat-soluble vitamin deficiencies and hypogammaglobulinaemia (Umar and Dibaise 2010). The presence of low serum albumin, elevated faecal α1-antitrypsin, low IgG and lymphopenia are in keeping with PLE. Most cases are secondary to underlying disorders, with protein leakage through either mucosal injury, for example in inflammatory bowel diseases (IBD) and menetrier’s disease, or through abnormalities of the lymphatic system, as in primary intestinal lymphangiectasia (PIL), congestive heart failure, or after Fontan procedure (Barbati et al. 2021; Chehade et al. 2006; Ostrow et al. 2006).

**Fig. 1:**
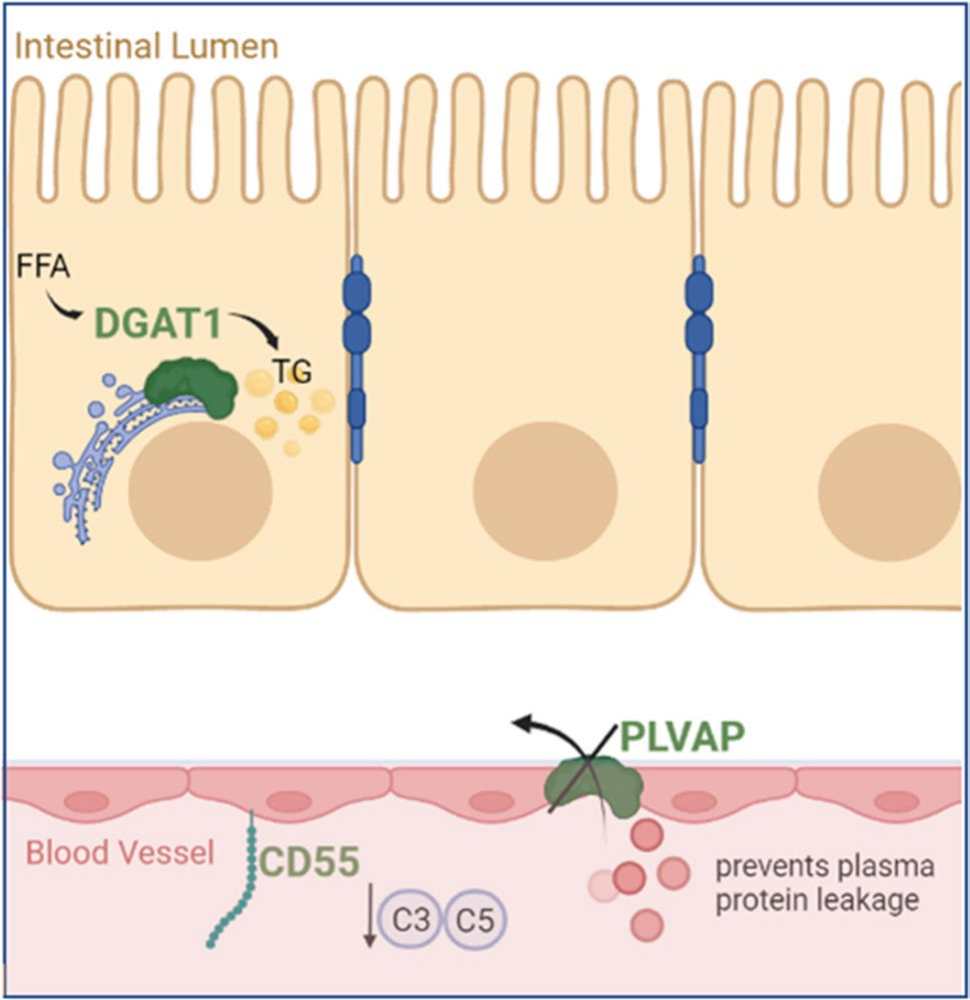
Cellular locations of defective proteins involved in primary PLE. *FFA* free fatty acids, *TG* triglycerides, *DGAT1* diacylglycerol acyltransferase, *PLVAP* plasmalemma vesicle-associated protein, *CD55* complement decay accelerating factor

Syndromic causes of PLE with intestinal lymphangiectasia include Hennekam syndrome with CCBE1 (Alders et al. 2009) or FAT4 mutations (Alders et al. 2014) and lymphedema–distichiasis syndrome caused by FOXC2 mutations (Vreeburg et al. 2008). In recent years, several rare non-syndromic genetic disorders causing PLE have been described. CD55 mutation has been reported to cause autosomal-recessive CHAPLE (**c**omplement **h**yperactivation, **a**ngiopathic thrombosis and **p**rotein-**l**osing **e**nteropathy) syndrome (Ozen et al. 2017). Mutations in acyl-CoA:diacylglycerol acyltransferase (DGAT) 1 are reported to cause congenital PLE owing to aberrant lipid metabolism (Stephen et al. 2016). While mutations in the plasmalemma vesicle-associated protein (PLVAP) were found to result in a very severe form of PLE characterised by hypoproteinaemia and hypertriglyceridaemia secondary to the deletion of the diaphragms of endothelial fenestrae (Elkadri et al. 2015). Here, we will discuss mutations in DGAT1, CD55 and PLVAP leading to PLE (Fig. 1)*.*

### DGAT1

Mutations in DGAT1 associated with PLE were first described in 2012 by Haas et al., to date there are less than 30 cases described. They detailed a non-consanguineous Ashkenazi Jewish family in which two of three siblings displayed early-onset diarrhoea with PLE and hypoalbuminaemia. They uncovered a homozygous splice-site mutation leading to exon 8 deletion, which yields a complete null allele with no expressed DGAT1 protein or activity (Haas et al. 2012). DGAT1 deficiency is a rare autosomal-recessive condition caused by loss-of-function mutations in the DGAT1 gene which encodes a diacylCoA:diacylglycerol acyl transferase that converts diacylglycerides to triglycerides (TGs) by adding an acyl CoA moiety. It plays a key role in enzymatic cellular diacylglycerol lipid metabolism, triglyceride synthesis and adipose tissue formation (Cases et al. 1998). In the small intestine, DGAT helps to absorb TGs, whereas in the liver, it produces TGs from fatty acids synthesised de novo or taken up from the circulation (Yen et al. 2008). DGAT has previously been investigated as a target for treating obesity. A DGAT1-knockout mouse model has shown that selective inhibition of DGAT-mediated triglyceride synthesis leads to altered triglyceride metabolism, increased energy expenditure and increased activity in terms of distance moved (Smith et al. 2000). Interestingly, pradigastat, a selective DGAT-1 inhibitor, had dose-related adverse events in a human trial, including mild to moderate diarrhoea, consistent with the DGAT1-deficient phenotype (Meyers et al. 2015).

DGAT1-deficient patients have largely presented within the first 3 months of life with early-onset non-bloody watery diarrhoea, emesis, hypoalbuminaemia and PLE. Growth failure, recurrent infections and the need for albumin infusions and parenteral nutrition have been commonly reported. While earlier cases reported elevated triglycerides, subsequent cases have had normal serum lipid profile, or reduced levels of high-density lipoprotein (Stephen et al. 2016). Initial studies indicated some loss of microvilli in duodenal biopsies; however, it is unclear if this persists in the absence of enteral lipids (van Rijn et al. 2018). DGAT1 deficiency has been mainly described in patients of Turkish, Chinese, Ashkenazi Jewish and Caucasian descent.

In addition to the splice-site mutation causing deletion of exon 8 (Haas et al. 2012), other mutations have also been described including missense (Gluchowski et al. 2017; Stephen et al. 2016), nonsense, homozygous recessive (van Rijn et al. 2018; Ye et al. 2019) and compound heterozygous mutations (Xu et al. 2020). In the missense mutations, residual enzyme activity is present and described cases are less severe than when a null mutation produces no enzyme. However, there is no clear genotype–phenotype correlation in DGAT1 deficiency, as patients from the same family with the same mutations have developed varied clinical characteristics ranging from complete resolution of GI symptoms to death secondary to malnutrition and sepsis.

Patient-derived intestinal organoids and dermal fibroblasts used in defining the molecular pathomechanism of DGAT1 deficiency show aberrant lipid metabolism including reduced lipid droplet and TG formation on incubation (van Rijn et al. 2018). In addition, DGAT1-deficient cells are shown to be more susceptible to lipid-induced toxicity, which may provide an explanation for the development of PLE in these patient. Dietary fat restriction has been shown to restore normal faecal protein clearance in DGAT1 deficiency which supports the concept that cellular lipotoxicity may be one of the driving forces for ongoing faecal protein loss in untreated PLE.

**Fig. 2:**
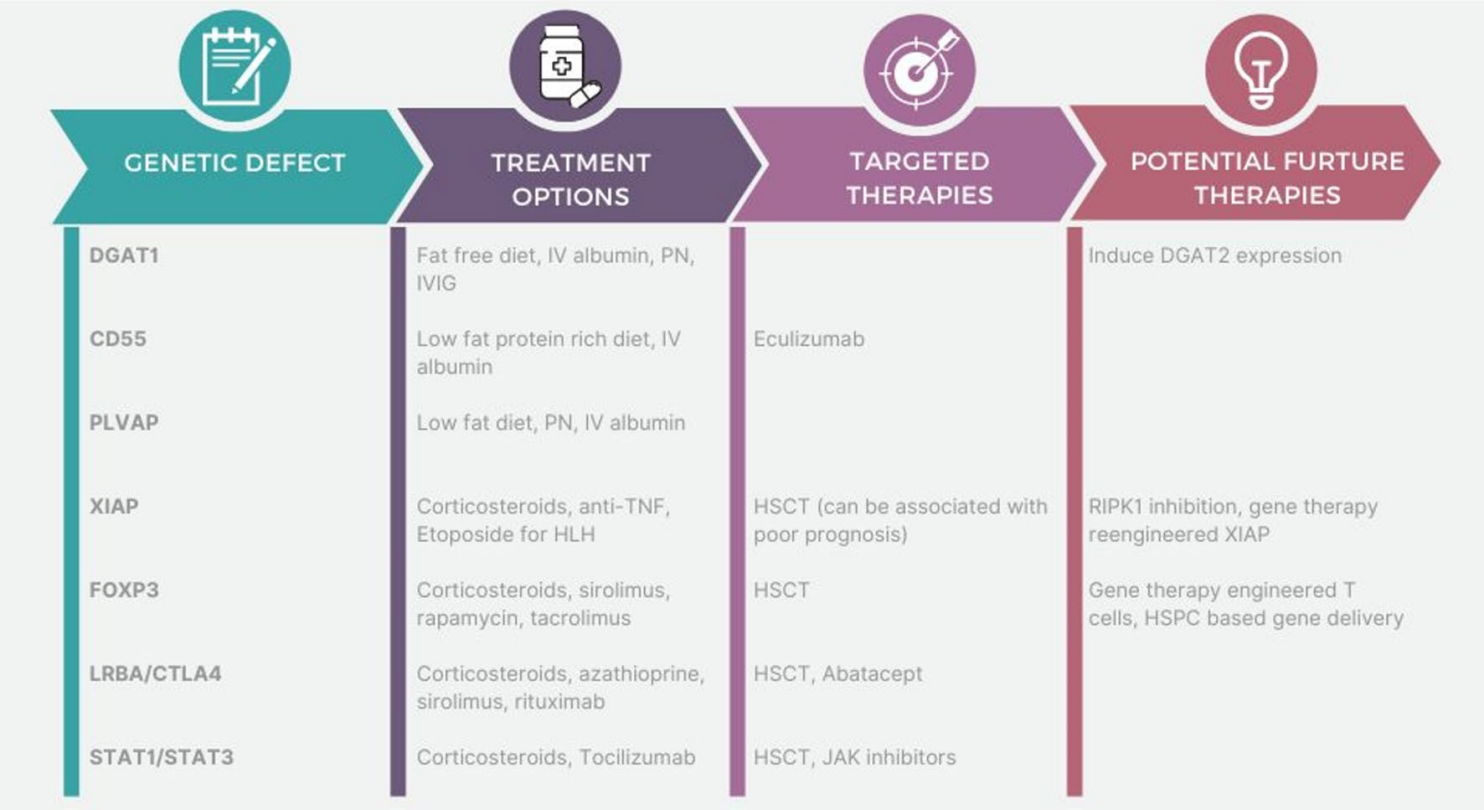
Enhanced understanding of the underlying genetics in PLE and AIE leads to mechanism-based therapies

Early introduction of a fat-restricted diet in these patients may prevent the development of more severe PLE. First-line therapy includes a fat-free diet with supplementation of fat-soluble vitamins and essential fatty acids. Intravenous administration of essential fatty acid is well tolerated, presumably as it bypasses absorption through the gut epithelium. Supportive therapy options include intravenous albumin and/or gamma globulin, micronutrient supplementation and parenteral nutrition (Fig. 2). Other therapies including cholestyramine and pancreatic enzymes have been described; however, due to small numbers, their therapeutic effect has not been established (van Rijn et al. 2018). Therapeutic strategies to induce DGAT2 expression might potentially provide an additional, viable treatment strategy in DGAT1 deficiency (van Rijn et al. 2018).

### CD55

CD55 deficiency is associated with **c**omplement **h**yperactivation, **a**ngiopathic thrombosis and **p**rotein-**l**osing **e**nteropathy (the CHAPLE, OMIM#226300). Ozen et al. in 2017 described 11 patients from 8 consanguineous families with PLE. They identified five distinct homozygous, loss of function CD 55 variants, with heterozygous persons being unaffected (Ozen et al. 2017). The complement system is an essential part of the innate immunity system requiring careful regulation. While complement deficiency can lead to susceptibility to certain infections, inappropriate activation of complement can also be a cause for disease. CD55 is a membrane-bound complement regulator that prevents the formation of new C3 and C5 convertases and accelerates their decay, it thereby guards host tissues from complement-induced damage during complement activation. CD55 encodes the decay accelerating factor, which is widely expressed in multiple tissues, including the gastrointestinal tract (Baines and Brodsky 2017; Kurolap et al. 2017). Similar to other complementopathies such as paroxysmal nocturnal haemoglobinuria (PNH) and atypical haemolytic uremic syndrome (aHUS), patients with CD55 deficiency have elevated membrane attack complex (MAC) deposition, possibly leading to the observed intestinal injury and facilitating the protein loss.

CD55-deficient patients invariably develop hypoproteinaemia together with oedema, vomiting, abdominal pain and diarrhoea (Kurolap et al. 2017; Ozen et al. 2017). Described patients exhibit micronutrient deficiencies, anaemia, growth failure and recurrent infections. A significant life-threatening risk of recurring thrombotic events has been reported in 40–50% of those affected. Reported hypercoagulability resulted in central line thromboses, Budd Chiari syndrome and superior vena cava syndrome (Kurolap et al. 2017, 2019; Ozen et al. 2017). The pro-thrombotic state in PLE patients may be attributed to enteric loss of anticoagulation factors and hypoalbuminaemia. Histology shows extensive lymphangiectasia, although inflammation similar to that seen in inflammatory bowel disease has also been observed. Age of onset of symptoms is considerably later than in DGAT1 deficiency, with the majority presenting in early childhood before age 6 years and the youngest presenting at 10 months. Additionally, there is a report of adult onset PLE, starting at the age of 25 years, previously treated as Crohns, who later developed superior mesenteric vein thrombosis. Targeted CD55 sequencing revealed a novel homozygous nonsense variant (Hagin et al. 2021). Among described cases, there is phenotypic heterogeneity, ranging from death due to thrombotic events and minimally symptomatic homozygotes. Disease expression may be modulated by other complement proteins, together with other genetic and environmental modifiers including diet and microbiome composition (Ozen et al. 2021). CD55 deficiency occurs globally but predominates in areas of high consanguinity such as the Igdir region of Turkey and the Bukharan Jewish population, with a carrier frequency of 1:17 (Kurolap et al. 2022; Ozen et al. 2021).

Treatment includes albumin infusion, vitamin and micronutrient supplementation and a low-fat protein-rich diet with medium-chain TG. Recently, the effective use of eculizumab has been reported in these patients (Fig. 2). Eculizumab is a humanised anti-C5 monoclonal antibody and terminal complement inhibitor. It is previously established as a treatment in other complementopathies such as aHUS and PNH (Hillmen et al. 2006; Legendre et al. 2013). Eculizumab led to significant reduction in MAC deposition on leukocytes from treated patients with low levels maintained over time (Kurolap et al. 2019). Cessation of gastrointestinal pathology was observed together with restoration of normal immunity and metabolism (Ozen et al. 2021). In addition, patients were able to stop a low-fat diet. Eculizumab treatment should be lifelong, immediate flare-up of symptoms was observed when the medication was withdrawn (Kurolap et al. 2019; Ozen et al. 2021). Upstream blockade at the C3 level or a combination of C3 and C5 blockade could be a potential future avenue of research, especially in patients with ongoing thrombocytosis or thrombosis (Ozen et al. 2021).

### PLVAP

Mutation in the plasmalemma vesicle-associated protein (PLVAP) gene results in a distinct severe form of PLE characterised by hypoproteinaemia, hypoalbuminaemia, and hypertriglyceridaemia (Elkadri et al. 2015). It was first described in 2015 in a neonate of Afghan descent with consanguineous parents, resulting in a severe fatal sieving hypoproteinaemia and enteropathy. There have been four further published cases. PLVAP encodes endothelial specific type II integral membrane protein-forming homodimers. It is essential in forming diaphragms of endothelial fenestrae and lymphatic capillaries (Stan et al. 2012). In the intestine, it plays a central role in the absorption of interstitial molecules into the blood or lymphatics by providing a filtration system to maintain blood, lymph and tissue homeostasis (Elkadri et al. 2015). Deletion of fenestral diaphragms causes leakage of plasma proteins into the interstitium of organs with fenestrated capillaries such as intestine, pancreas and adrenals (Rantakari et al. 2015; Stan et al. 2012).

The original identified PLVAP nonsense mutation (c.1072C > T p.Arg358*) introduces a premature stop codon in exon 3 (Elkadri et al. 2015). Subsequent described novel stop mutations, both nonsense (c.988C > T, p.Q330*) and frameshift (c.339dupT; p.Ala114Cysfs9), have similarly resulted in fatal disease (Broekaert et al. 2018; Gorukmez et al. 2019). The described nonsense mutations (p.Arg358 andp.Gln330) truncated the protein in the second coiled-coil domain while the frameshift mutation is predicted to truncate the protein in the N-glycosylation sites of the extracellular domain. A homozygous missense variant in two kindred cases, occurring in the first exon of PLVAP (c.101 T > C;p.Leu34Pro) and compatible with residual PLVAP function, resulted in a milder variant with later onset (Kurolap et al. 2018). All described cases to date occurred in consanguineous offspring of Turkish, Afghani or Arabic descent.

Patients invariably presented with diarrhoea and hypoalbuminaemia. Those with stop-codon mutations presented early in life and had associated anomalies including dysmorphism, cardiac, iris and renal anomalies. In addition to profound hypalbuminaemia, hypertriglyceridaemia, hypothyroidism and venous thrombosis was also evident (Broekaert et al. 2018; Elkadri et al. 2015; Gorukmez et al. 2019). Electron microscopy of intestinal biopsy samples in the index case demonstrated a complete lack of diaphragms in the fenestrae and caveolae of endothelial cells of all the capillaries in the duodenum villi (Elkadri et al. 2015). The pathogenesis of PLE differs from that found in CD55 deficiency as there is no evidence of inflammation or abnormalities of the lymphatic system. Instead, the defect lies in endothelial cells, with protein loss due to leaky fenestrated capillaries in the intestine. The kindred patients with the reported missense variant presented later at age 22 years and 2.5 years with anasarca, severe hypalbuminaemia and hypogammaglobulinaemia. There was partial penetrance of hypertriglyceridaemia and there were no extraintestinal manifestations. Electron microscopy of duodenal biopsies revealed preserved endothelial fenestral diaphragms. They were successfully treated with low-fat medium-chain TG diet (Kurolap et al. 2018) (Fig. 2). Despite treatment with parenteral nutrition and albumin infusions, all cases of stop-codon mutation resulted in fatality (Broekaert et al. 2018; Elkadri et al. 2015; Gorukmez et al. 2019).

PLVAP mutations represent a very rare cause of PLE, genetic testing for PLVAP should be considered in the work-up of congenital diarrhoeal disorders. Further research in animals modelling the mechanisms of PLVAP downregulation is needed to elucidate interventional strategies.

## XIAP

X-linked inhibitor of apoptosis (XIAP) deficiency also known as X-linked lymphoproliferative syndrome type 2, (XLP-2) is a rare inborn error of immunity. It was first described in 2006 by Riguad et al. in patients from three families (Rigaud et al. 2006). It is characterised by immune dysregulation and clinical manifestations, including recurrent haemophagocytic lymphohistiocytosis (HLH), IBD (resembling Crohns phenotype), hypogammaglobulinaemia, susceptibility to infections, splenomegaly and cytopaenias. XIAP deficiency is a rare inborn error of immunity caused by mutations in the XIAP/BIRC4gene, which consists of six coding exons. XIAP is a ubiquitin ligase that ubiquitinates receptor-interacting protein (RIP) kinases and controls signalling of intracellular pathogen recognition receptors as well as cytokine receptors (Mudde et al. 2021). XIAP is expressed in multiple cell types of the innate and adaptive immune system as well as in the epithelial cell compartment. To date, over 90 disease-causing mutations have been described, including nonsense, missense, frameshift and inotronic mutations (Mudde et al. 2021). Absence of XIAP protein generally occurs in the case of nonsense mutations and deletions, residual expression of protein may occur in missense and splice-site mutations. Similar to previously described genes, there is no definite correlation between genotype and phenotype, with large variability observed in affected siblings (Kim 2018). Female carriers are generally asymptomatic; however, there are reports of female carriers manifesting colitis symptoms (Aguilar and Latour 2015).

Gastrointestinal manifestations of XIAP include early onset of colitis and failure to thrive (Speckmann et al. 2013). IBD resembles Crohn’s disease clinically and histologically and is frequently severe and refractory to standard therapies. Patients may suffer from severe perianal fistulae, recurrent strictures and abscesses (Zeissig et al. 2015). Epithelioid granulomas and crypt abscesses can be seen on histology anywhere throughout the GI tract. Age of onset varies widely, from the neonatal period into adulthood (Aguilar and Latour 2015). IBD is observed only in one-third of XIAP-deficient patients. HLH and splenomegaly are the most frequently observed traits (54% and 57%, respectively) (Aguilar and Latour 2015). EBV is the major trigger for HLH; however, cytomegalovirus and human herpes virus 6 have also been implicated.

Treatment generally consists of immunosuppression including corticosteroids, anti-TNF for IBD and etoposide for HLH (Fig. 2). HSCT offers a curative treatment; however, can be associated with a poor prognosis. Mortality rates post HSCT have been reported as high as 63%, with some patients developing severe inflammatory complications following HSCT (Marsh et al. 2013). Potential future treatment options include gene therapy where the phagocyte and lymphocyte defects are corrected by reengineered XIAP but epithelial defects remain (Azabdaftari and Uhlig 2021). Therapies that target RIP kinases might have potential in the treatment of intestinal inflammation considering that inhibition of RIPK1 rescued Paneth cell loss in XIAP-deficient mice (Strigli et al. 2021).

## Autoimmune enteropathy

**Fig. 3:**
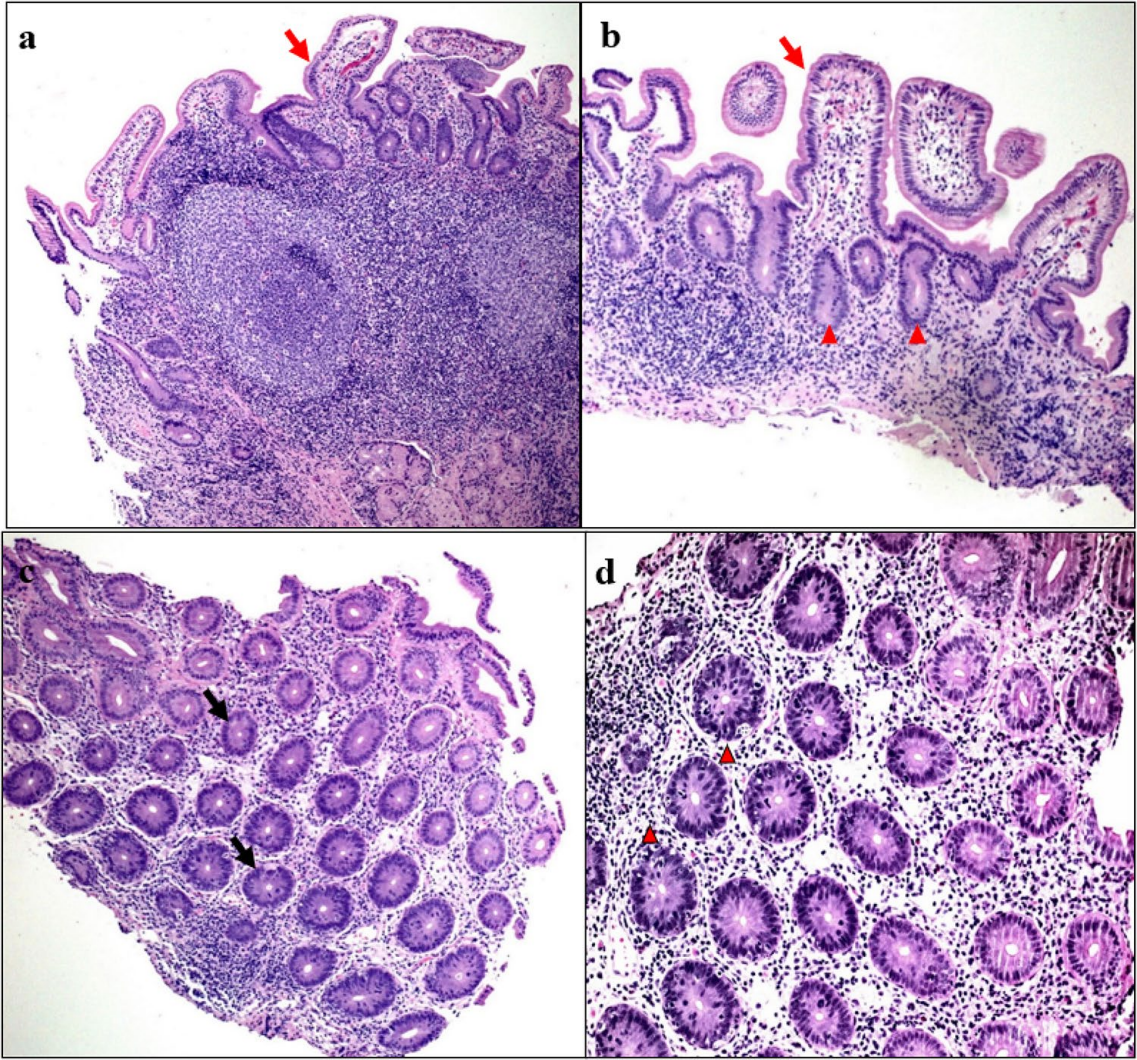
Duodenum, terminal ileum and colonic biopsies from a patient with IPEX- like syndrome due to LRBA deficiency. **a** and **b** Duodenal and terminal ileal biopsies (40 × magnification) demonstrating complete absence of goblet (arrows) and Paneth cells (arrow heads). No other abnormality was noted. **c** and **d** Colonic biopsy from the sigmoid shows complete loss of goblet cells (arrows), numerous apoptotic bodies (arrow heads) and increased intraepithelial lymphocytes in the crypts (40 × and 100 × magnifications, respectively)

Autoimmune enteropathy (AIE) is an immune-mediated multifaceted disorder presenting with intractable diarrhoea predominantly in early childhood (Ahmed et al. 2019). Although initially thought to be a disease of childhood, an increasing number of adult onset cases have been reported (Akram et al. 2007; van Wanrooij et al. 2021). Unsworth and Walker-Smith proposed the initial diagnostic criteria in 1985 which included severe diarrhoea unresponsive to dietary changes, evidence of autoimmunity in the absence of a known immunodeficiency (Unsworth and Walker-Smith 1985). More recently, Akram et al. proposed a new set of criteria for adults, consisting of diarrhoea greater than 6 weeks duration, malabsorption and villous blunting on small bowel biopsy (Akram et al. 2007). AIE occurs predominantly in males with an average age of presentation less than 6 months (Ahmed et al. 2019). The classical symptoms consist of severe, hyper-secretory non-bloody diarrhoea, malabsorption, electrolyte abnormalities and failure to thrive (Chen et al. 2020). There is frequently extraintestinal involvement, with many patients exhibiting hypothyroidism, arthritis, autoimmune hepatitis and diabetes mellitus (Ahmed et al. 2019). Up to 83% of patients with AIE may have one or more other autoimmune disorders (Ahmed et al. 2019). Endoscopic findings can be categorised into four main histological patterns; active duodenitis, celiac-like (villous atrophy, glandular crypt hyperplasia, and marked increase in T lymphocytes in the surface epithelium), graft versus host-like and mixed pattern (Masia et al. 2014) (Fig. 3). The largest case series of AIE which included 40 paediatric and adult patients found that celiac-like pattern was most prevalent at 50% (Villanacci et al. 2019).

The exact pathogenesis of AIE remains unclear. Abnormal expression of self-antigens on epithelial cells activates CD4 T lymphocytes, which produces downstream effects eventually leading to the destruction of the enterocytes through apoptosis or other cytotoxic effects (Wosen et al. 2018). Autoantibodies to intestinal cells have also been implicated; however; are not always diagnostic and can be found in other conditions such as inflammatory bowel disease and human immunodeficiency virus (Chen et al. 2020).

The main syndromes classified under autoimmune enteropathy include immunodysregulation polyendocrinopathy enteropathy X-linked (IPEX) syndrome due to mutations in FOXP3, IPEX-like syndromes that have the features of IPEX but do not have the FOXP3 mutation (includes mutations in BACH2, CD25, STAT1, STAT3, CTLA-4, ITCH, MALT1, PTPN2 and LRBA) and autoimmune polyglandular syndrome type 1 (APS-1) due to mutations in the AIRE gene. Here, we will discuss FOXP3 and gene mutations in LRBA, CTLA-4, STAT1 and STAT3 leading to IPEX-like syndromes.

### IPEX

Mutations in the transcription factor forkhead box P3 (FOXP3) gene lead to IPEX syndrome (Fontenot et al. 2005). IPEX is an X-linked recessive autoimmune disease caused by dysfunction of regulatory T (Treg) cells. It primarily affects males, due to loss-of-function mutations in the FOXP3 gene located on the X chromosome. IPEX syndrome is inherited in boys in an X-linked recessive manner. The FOXP3 gene is located in the centromeric region of X chromosome Xp11.23–Xq13.3. Mutations of this gene lead to defects in the DNA-binding domain (forkhead domain). The abnormal protein cannot bind to its binding spot on the DNA, impairing the development and function of T regulatory cells, leading to a loss of CD4 + CD25 + T regulatory cells and uncontrolled proliferation of activated CD4 + effector cells (Fontenot et al. 2017). This results in abnormal immune responses causing autoimmune manifestations such as enteropathy, endocrinopathies and eczema (Murguia-Favela et al. 2014). In a murine IPEX model, normal Treg cells were found to rescue disease manifestations, showing that lack of functional Treg cells is the primary cause of disease development (Fontenot et al. 2005). Over 70 novel mutations of the FOXP3 gene have been identified; however, genotype–phenotype correlation remains uncertain (Bacchetta et al. 2016). The most common mutations occur in the forkhead domain followed by the repressor and leucine-zipper domains. The more severe forms of IPEX resulting in neonatal death tend to occur in patients with frameshift mutations and in-frame deletions (Ben‐skowronek 2021).

The majority of patients have disease onset within the first year of life, with a median age of 2 months in retrospective, multicentre study (Barzaghi et al. 2018). IPEX manifests with severe multiple autoimmunities, particularly the skin (severe dermatitis), gastrointestinal tract (enteritis with refractory diarrhoea), blood (haemolytic anaemia, thrombocytopaenia, neutropaenia), renal (nephropathy) and endocrinopathies (type 1 diabetes, thyroiditis, adrenal insufficiency) (Gambineri et al. 2018; Huang et al. 2020). The presence of autoantibodies helps to support the diagnosis of autoimmune enteropathy, although they may be absent. Singhi et al. found that 79% of paediatric patients had positive anti-enterocyte (AE) antibodies (Singhi et al. 2014). While Akram et al.’s study of 15 adult patients showed 27% with anti-goblet (AG) cell antibodies and 60% with AE antibodies (Akram et al. 2007). Additionally, autoantibodies including antinuclear antibody (ANA), anti-liver/kidney microsomal (anti-LKM) antibodies and anti-smooth muscle antibodies (anti-SMA) may be positive (Chen et al. 2020). Absence of CD4 + CD25 + regulatory T cells can be seen on flow cytometry of peripheral blood cells. Endoscopy may be macroscopically normal, although mucosal hyperaemia and ulcerations can be seen with the duodenum, the most common site of GI involvement (Villanacci et al. 2019). Small bowel villous blunting, loss of goblet and absence of Paneth cells and crypt apoptotic bodies can be seen (Masia et al. 2014). Sequencing for the FOXP3 gene is required for definitive diagnosis (Gambineri et al. 2018).

Treatment of AIE includes corticosteroids, with a reported response rate of 26% to steroids alone (Ahmed et al. 2019). Many patients will require additional immunosuppressive therapy and/or parenteral nutrition. Ahmed et al. found that 45% of patients with AIE required parenteral nutrition at some point (Ahmed et al. 2019). Barzaghi et al. reported immunosuppressive therapy use in 35%. Several studies have shown sirolimus to be superior to calcineurin inhibitors (Bacchetta et al. 2016). Recently, rapamycin has been shown to be clinically effective by supressing peripheral T cells while sparing Treg cells expressing wild-type FOXP3 (Passerini et al. 2020). Hematopoietic stem cell transplantation (HSCT) is the definitive treatment for IPEX and may be potentially curative (Chen et al. 2020) (Fig. 2). HSCT was reported to occur in 60% of 96 patients with a mean age at disease onset of 2 months (Barzaghi et al. 2018). Gambineri et al. reported a 10-year survival rate of 72.8% for those who underwent HSCT versus 57.3% who did not (Gambineri et al. 2018). Outcomes from HSCT are more favourable in the absence of multiorgan involvement prior to transplant (Barzaghi et al. 2018).

Current treatment options present limitations. There are currently two main gene-therapy-based approaches under development for the treatment of IPEX. These include engineered T cells that acquire regulatory function upon enforced FOXP3 expression (Charbonnier et al. 2019) and HSPC-based gene delivery using lentiviral or CRISPR-based approaches (Borna et al. 2022; Goodwin et al. 2020). The lifespan of transferred Treg cells in patients remains to be determined; hence, a gene-transfer-based approach may be necessary to cure disease.

### IPEX-like syndromes

Mutations in lipopolysaccharide responsive and beige-like anchor protein (LRBA) and cytotoxic T lymphocyte antigen 4 (CTLA4) lead to IPEX-like syndrome. The LRBA gene plays a pivotal role in the intracellular trafficking of cytotoxic T lymphocyte antigen 4 (CTLA4), by co-localising with CTLA4 in endosome vesicles, controlling its turnover (Lo et al. 2016). CTLA4 acts as a checkpoint for the immune system, inhibiting activation and expansion of self-reactive T cells and establishing peripheral tolerance (Schubert et al. 2014). It competes with CD28 for their shared ligands, CD80 and CD86, and removes them from antigen-presenting cells (APCs) via transendocytosis, thereby preventing continuous activation of T cells. LRBA deficiency results in very low CTLA4 expression resulting in overlap between these phenotypes (Schubert et al. 2014). Moreover, LRBA deficiency typically leads to lower levels of CTLA-4 than CTLA- 4 haploinsufficiency; therefore, these patients frequently present earlier in life (Hou et al. 2017). Treg cells are reduced in number and function in those with LRBA deficiency leading to similar clinical features as seen in IPEX (Charbonnier et al. 2015; Gámez-Díaz et al. 2016). While loss-of-function mutations in CTLA-4 results in T cell proliferation and abnormal Treg cell function (Schubert et al. 2014).

Described mutations in LRBA are located throughout the whole gene, with the majority of variants being missense, splice-site, indels or nonsense mutations (Pfundt et al. 2017). Rare cases of uniparental isodisomy have been described (Soler-Palacín et al. 2018). The majority of described mutations result in a complete loss of LRBA protein; however, some show residual expression (Ahmed et al. 2019). LRBA deficiency (OMIM#614700) is inherited in an autosomal-recessive pattern. Genetic studies confirming biallelic mutations in LRBA are the gold standard for diagnosis, there have, however, been reported cases of failure of whole-exome sequencing (WES) to reveal a genetic aberration which was subsequently revealed through whole-genome sequencing (WGS) (Merico et al. 2021).

In contrast, CTLA-4 haploinsufficiency is inherited in an autosomal-dominant manner with reported clinical penetrance of 60–67% (Jamee et al. 2021; Schwab et al. 2018). The CTLA-4 gene (OMIM#123890) is located on ch2q33.2. More than 70 distinct mutations have been described including missense, insertion/deletion frameshift, nonsense, splice-site and start-codon mutation (Jamee et al. 2021).

LRBA-deficient patients commonly present in infancy with colitis, recurrent infections (frequently sinopulmonary), lymphadenopathy, enlarged spleen, autoimmune cytopenias and endocrinopathies. While those with mutations in CTLA-4 tend to present later. In a cohort study of 76 patients with LRBA deficiency, the median age of onset of symptoms was 2 years (range, birth—25 years) (Tesch et al. 2020). In a worldwide cohort study of CTLA-4 mutation carriers, the median age of onset of symptoms was 11 years (Schwab et al. 2018). The hallmarks of disease are similar in LRBA and CTLA4 mutations and include hypogammaglobulinaemia, lymphoproliferation and autoimmunity (Lopez-Herrera et al. 2012). Immune dysregulation, organomegaly and recurrent infections are the most commonly reported manifestations (Gámez-Díaz et al. 2016). Autoimmune enteropathy is more common in LRBA deficiency, while autoimmune cutaneous disorders are seen more frequently in CTLA4 haploinsufficiency (Jamee et al. 2021). Lung involvement and autoimmune cytopaenia’s significantly correlate with fatal disease outcomes (Tesch et al. 2020). There is a reported increased risk of malignancy in LRBA deficiency, Tesch et al.’s study found that, over a 10-year follow-up, there was a 3.9% risk of malignancy (Tesch et al. 2020). Similarly Schwab et al. found an increased risk of lymphoma and gastric cancer in patients with mutated CTLA4 (Schwab et al. 2018). While the majority of patients have normal T cell counts, Gamez-Diaz et al., found that 73% of LRBA-deficient patients had reduced Treg cell numbers (Gámez-Díaz et al. 2016). The phenotype has a wide range, from asymptomatic to fatal disease (Kostel Bal et al. 2017).

Multiple different therapeutic strategies have been employed in the treatment of these IPEX-like syndromes including corticosteroids, IVIG, azathioprine, sirolimus, infliximab and rituximab (Gámez-Díaz et al. 2016; Jamee et al. 2021; Kostel Bal et al. 2017). HSCT has been used in severe disease and can be curative (Kiykim et al. 2019). In a systematic review of 434 patients with CTLA-4 haploinsufficiency or LRBA deficiency, 12.7% underwent HSCT, the overall survival rate in LRBA deficiency was lower than in CTLA-4 patients (Jamee et al. 2021). More recently, the CTLA4-Ig fusion protein, Abatacept, has emerged as an effective treatment strategy in both CTLA-4 haploinsufficiency and LRBA deficiency (Kiykim et al. 2019; Kostel Bal et al. 2017) (Fig. 2). Abatacept mimics the function of the cellular CTLA4 pool, in negatively regulating the immune responses by blockading or capturing CD80/86 molecules found on antigen-presenting cells (Alroqi et al. 2018; Kostel Bal et al. 2017).

In addition to mutations in LRBA and CTLA-4, overactivation of the JAK-STAT pathway leads to an IPEX-like phenotype caused by gain-of-function (GOF) mutations in signal transducer and activator of transcription 1 (STAT1) and STAT3. STAT1 mutations impair helper T cell (TH) response, particularly TH17, and are associated with an increased risk of infections, especially mucocutaneous candidiasis (Carey et al. 2019). Combined immunodeficiency, autoinflammation, enteropathy and autoimmunity are also described (Toubiana et al. 2016). Reduced Th17 populations can be seen on flow cytometry (Uzel et al. 2013). STAT3 GOF mutations are associated with lymphadenopathy, hepatosplenomegaly and autoimmunity including cytopaenias, hepatitis, enteropathy, diabetes mellitus and hypothyroidism (Flanagan et al. 2014). Ruxolitinib and baricitinib, oral JAK 1/2 inhibitors, along with tofacitinib, JAK 1/3 inhibitor, have been reported to improve disease control (Forbes et al. 2018; Higgins et al. 2015; Meesilpavikkai et al. 2018) (Fig. 2), including a case of ruxoltinib inducing complete remission (Parlato et al. 2019). There have also been reports of worsening of fungal infections as a complication of treatment (Forbes et al. 2018; Zimmerman et al. 2017). Additionally ruxolitinib has been used as a bridge to HSCT (Kayaoglu et al. 2021).

## Conclusion

Patients with early-onset diarrhoeal disorders pose a clinical and diagnostic challenge. A large number of potential aetiologies exist; however, advances in genetic sequencing have revealed many monogenic disorders. Early genetic testing in patients with infantile diarrhoeas should improve clinical care and prognostication as well as guiding management. Through a greater understanding of the underlying genetics, targeted therapies can be employed in these patients, including the use of curative HSCT. Gene therapy such as lentiviral or CRISPR-based approaches may be a viable future treatment strategy in many of these monogenic disorders but require further research and advances. Similarly, the evolving understanding of environmental factors and their influence on genotype–phenotype will likely enhance our understanding of the pathogenesis and treatment of these disorders.

## Data Availability

Data sharing does not apply to this article as no datasets were generated for this review.

## References

[CR1] Aguilar C, Latour S (2015). X-linked inhibitor of apoptosis protein deficiency: more than an X-linked lymphoproliferative syndrome. J Clin Immunol.

[CR2] Ahmed Z, Imdad A, Connelly JA, Acra S (2019). Autoimmune enteropathy: an updated review with special focus on stem cell transplant therapy. Dig Dis Sci.

[CR3] Akram S, Murray JA, Pardi DS, Alexander GL, Schaffner JA, Russo PA, Abraham SC (2007). Adult autoimmune enteropathy: Mayo Clinic Rochester experience. Clin Gastroenterol Hepatol.

[CR4] Alders M, Hogan BM, Gjini E, Salehi F, Al-Gazali L, Hennekam EA, Holmberg EE, Mannens MMAM, Mulder MF, Johan Offerhaus GA, Prescott TE, Schroor EJ, Verheij JBGM, Witte M, Zwijnenburg PJ, Vikkula M, Schulte-Merker S, Hennekam RC (2009). Mutations in CCBE1 cause generalized lymph vessel dysplasia in humans. Nat Genet.

[CR5] Alders M, Al-Gazali L, Cordeiro I, Dallapiccola B, Garavelli L, Beyhan T, Salehi F, Haagmans MA, Mook OR, Majoie CB, Mannens MM, Hennekam RC (2014). Hennekam syndrome can be caused by FAT4 mutations and be allelic to Van Maldergem syndrome. Hum Genet.

[CR6] Alroqi FJ, Charbonnier LM, Baris S, Kiykim A, Chou J, Platt CD, Algassim A, Keles S, Al Saud BK, Alkuraya FS, Jordan M, Geha RS, Chatila TA (2018). Exaggerated T follicular helper cell responses in LRBA deficiency due to failure of CTLA4-mediated regulation. J Allergy Clin Immunol.

[CR7] Azabdaftari A, Uhlig HH (2021). Paneth cell dysfunction and the intestinal microbiome in XIAP deficiency. Sci Immunol.

[CR8] Bacchetta R, Barzaghi F, Roncarolo MG (2016). From IPEX syndrome to FOXP3 mutation: a lesson on immune dysregulation. Ann N Y Acad Sci.

[CR9] Baines AC, Brodsky RA (2017). Complementopathies. Blood Rev.

[CR10] Barbati F, Marrani E, Indolfi G, Lionetti P, Trapani S (2021). Menetrier disease and Cytomegalovirus infection in paediatric age: report of three cases and a review of the literature. Eur J Pediatr.

[CR11] Barzaghi F, Amaya Hernandez LC, Neven B, Ricci S, Kucuk ZY, Bleesing JJ, Nademi Z, Slatter MA, Ulloa ER, Shcherbina A, Roppelt A, Worth A, Silva J, Aiuti A, Murguia-Favela L, Speckmann C, Carneiro-Sampaio M, Fernandes JF, Baris S (2018). Long-term follow-up of IPEX syndrome patients after different therapeutic strategies: an international multicenter retrospective study. J Allergy Clin Immunol.

[CR12] Ben-skowronek I (2021). IPEX syndrome: genetics and treatment options. Genes.

[CR13] Borna S, Lee E, Sato Y, Bacchetta R (2022). Towards gene therapy for IPEX syndrome. Eur J Immunol.

[CR14] Braamskamp MJAM, Dolman KM, Tabbers MM (2010). Clinical practice Protein-losing enteropathy in children. Eur J Pediatr.

[CR15] Broekaert IJ, Becker K, Gottschalk I, Körber F, Dötsch J, Thiele H, Altmüller J, Nürnberg P, Hünseler C, Cirak S (2018). Mutations in plasmalemma vesicle-associated protein cause severe syndromic protein-losing enteropathy. J Med Genet.

[CR16] Carey B, Lambourne J, Porter S, Hodgson T (2019). Chronic Mucocutaneous candidosis due to gain-of-function mutation in STAT1. Oral Dis.

[CR17] Cases S, Smith SJ, Zheng YW, Myers HM, Lear SR, Sande E, Novak S, Collins C, Welch CB, Lusis AJ, Erickson SK, Farese RV (1998). Identification of a gene encoding an acyl CoA:diacylglycerol acyltransferase, a key enzyme in triacylglycerol synthesis. Proc Natl Acad Sci USA.

[CR18] Charbit-Henrion F, Parlato M, Hanein S, Duclaux-Loras R, Nowak J, Begue B, Rakotobe S, Bruneau J, Fourrage C, Alibeu O, Rieux-Laucat F, Lévy E, Stolzenberg MC, Mazerolles F, Latour S, Lenoir C, Fischer A, Picard C, Aloi M (2018). Diagnostic yield of next-generation sequencing in very early-onset inflammatory bowel diseases: a multicentre study. J Crohn’s Colitis.

[CR19] Charbonnier LM, Janssen E, Chou J, Ohsumi TK, Keles S, Hsu JT, Massaad MJ, Garcia-Lloret M, Hanna-Wakim R, Dbaibo G, Alangari AA, Alsultan A, Al-Zahrani D, Geha RS, Chatila TA (2015). Regulatory T cell deficiency and immune dysregulation, polyendocrinopathy, enteropathy, X-linked-like disorder due to loss of function mutations in LRBA. J Allergy Clin Immunol.

[CR20] Charbonnier L-M, Cui Y, Stephen-Victor E, Harb H, Lopez D, Bleesing JJ, Garcia-Lloret MI, Chen K, Ozen A, Carmeliet P, Li MO, Pellegrini M, Chatila TA (2019). Functional reprogramming of regulatory T cells in the absence of Foxp3. Nat Immunol.

[CR21] Chehade M, Magid MS, Mofidi S, Nowak-Wegrzyn A, Sampson HA, Sicherer SH (2006). Allergic eosinophilic gastroenteritis with protein-losing enteropathy: intestinal pathology, clinical course, and long-term follow-up. J Pediatr Gastroenterol Nutr.

[CR22] Chen CB, Tahboub F, Plesec T, Kay M, Radhakrishnan K (2020). A review of autoimmune enteropathy and its associated syndromes. Dig Dis Sci.

[CR23] Crowley E, Warner N, Pan J, Khalouei S, Elkadri A, Fiedler K, Foong J, Turinsky AL, Bronte-Tinkew D, Zhang S, Hu J, Tian D, Li D, Horowitz J, Siddiqui I, Upton J, Roifman CM, Church PC, Wall DA (2020). Prevalence and clinical features of inflammatory bowel diseases associated with monogenic variants, identified by whole-exome sequencing in 1000 children at a single center. Gastroenterology.

[CR24] Elkadri A, Thoeni C, Deharvengt SJ, Murchie R, Guo C, Stavropoulos JD, Marshall CR, Wales P, Bandsma RHJ, Cutz E, Roifman CM, Chitayat D, Avitzur Y, Stan RV, Muise AM (2015). Mutations in plasmalemma vesicle associated protein result in sieving protein-losing enteropathy characterized by hypoproteinemia, hypoalbuminemia, and hypertriglyceridemia. CMGH.

[CR25] Flanagan SE, Haapaniemi E, Russell MA, Caswell R, Allen HL, de Franco E, Mcdonald TJ, Rajala H, Ramelius A, Barton J, Heiskanen K, Heiskanen-Kosma T, Kajosaari M, Murphy NP, Milenkovic T, Seppänen M, Lernmark Å, Mustjoki S, Otonkoski T (2014). Activating germline mutations in STAT3 cause early-onset multi-organ autoimmune disease. Nat Genet.

[CR26] Fontenot JD, Rasmussen JP, Williams LM, Dooley JL, Farr AG, Rudensky AY (2005). Regulatory T cell lineage specification by the forkhead transcription factor Foxp3. Immunity.

[CR27] Fontenot JD, Gavin MA, Rudensky AY (2017). Foxp3 programs the development and function of CD4+CD25+ regulatory T cells. J Immunol.

[CR28] Forbes LR, Vogel TP, Cooper MA, Castro-Wagner J, Schussler E, Weinacht KG, Plant AS, Su HC, Allenspach EJ, Slatter M, Abinun M, Lilic D, Cunningham-Rundles C, Eckstein O, Olbrich P, Guillerman RP, Patel NC, Demirdag YY, Zerbe C (2018). Jakinibs for the treatment of immunodysregulation in patients with gain of function STAT1 or STAT3 mutations. J Allergy Clin Immunol.

[CR29] Gambineri E, Mannurita SC, Hagin D, Vignoli M, Anover-Sombke S, Deboer S, Segundo GRS, Allenspach EJ, Favre C, Ochs HD, Torgerson TR (2018). Clinical, immunological, and molecular heterogeneity of 173 patients with the phenotype of immune dysregulation, polyendocrinopathy, enteropathy, X-linked (IPEX) syndrome. Front Immunol.

[CR30] Gámez-Díaz L, August D, Stepensky P, Revel-Vilk S, Seidel MG, Noriko M, Morio T, Worth AJJ, Blessing J, van de Veerdonk F, Feuchtinger T, Kanariou M, Schmitt-Graeff A, Jung S, Seneviratne S, Burns S, Belohradsky BH, Rezaei N, Bakhtiar S (2016). The extended phenotype of LPS-responsive beige-like anchor protein (LRBA) deficiency. J Allergy Clin Immunol.

[CR31] Gluchowski NL, Chitraju C, Picoraro JA, Mejhert N, Pinto S, Xin W, Kamin DS, Winter HS, Chung WK, Walther TC, Farese RV (2017). Identification and characterization of a novel DGAT1 missense mutation associated with congenital diarrhea. J Lipid Res.

[CR32] Goodwin M, Lee E, Lakshmanan U, Shipp S, Froessl L, Barzaghi F, Passerini L, Narula M, Sheikali A, Lee CM, Bao G, Bauer CS, Miller HK, Garcia-Lloret M, Butte MJ, Bertaina A, Shah A, Pavel-Dinu M, Hendel A (2020). CRISPR-based gene editing enables FOXP3 gene repair in IPEX patient cells. Sci Adv.

[CR33] Gorukmez O, Gorukmez O, Demiroren K (2019). Novel PLVAP mutation in protein losing enteropathy. Fetal Pediatr Pathol.

[CR34] Haas JT, Winter HS, Lim E, Kirby A, Blumenstiel B, DeFelice M, Gabriel S, Branski D, Grueter CA, Toporovski MS, Walther TC, Daly MJ, Farese RV (2012). DGAT1 mutation is linked to a congenital diarrheal disorder. J Clin Investig.

[CR35] Hagin D, Lahav D, Freund T, Shamai S, Brazowski E, Fishman S, Kurolap A, Baris Feldman H, Shohat M, Salomon O (2021). Eculizumab-responsive adult onset protein losing enteropathy, caused by germline CD55-deficiency and complicated by aggressive angiosarcoma. J Clin Immunol.

[CR36] Higgins E, al Shehri T, McAleer MA, Conlon N, Feighery C, Lilic D, Irvine AD (2015). Use of ruxolitinib to successfully treat chronic mucocutaneous candidiasis caused by gain-of-function signal transducer and activator of transcription 1 (STAT1) mutation. J Allergy Clin Immunol.

[CR37] Hillmen P, Young NS, Schubert J, Brodsky RA, Socié G, Muus P, Röth A, Szer J, Elebute MO, Nakamura R, Browne P, Risitano AM, Hill A, Schrezenmeier H, Fu C-L, Maciejewski J, Rollins SA, Mojcik CF, Rother RP, Luzzatto L (2006). The complement inhibitor eculizumab in paroxysmal nocturnal hemoglobinuria. N Engl J Med.

[CR38] Hou TZ, Verma N, Wanders J, Kennedy A, Soskic B, Janman D, Halliday N, Rowshanravan B, Worth A, Qasim W, Baxendale H, Stauss H, Seneviratne S, Neth O, Olbrich P, Hambleton S, Arkwright PD, Burns SO, Walker LSK, Sansom DM (2017). Identifying functional defects in patients with immune dysregulation due to LRBA and CTLA-4 mutations. Blood.

[CR39] Huang Q, Liu X, Zhang Y, Huang J, Li D, Li B (2020). Molecular feature and therapeutic perspectives of immune dysregulation, polyendocrinopathy, enteropathy, X-linked syndrome. Journal of Genetics and Genomics = Yi Chuan Xue Bao.

[CR40] Jamee M, Hosseinzadeh S, Sharifinejad N, Zaki-Dizaji M, Matloubi M, Hasani M, Baris S, Alsabbagh M, Lo B, Azizi G, Allergy P, Jeffrey Modell I (2021). Comprehensive comparison between 222 CTLA-4 haploinsufficiency and 212 LRBA deficiency patients: a systematic review. Clin Exp Immunol.

[CR41] Kayaoglu B, Kasap N, Surucu Yilmaz N, Charbonnier LM, Geckin B, Akcay A, Bilgic Eltan S, Ozturk G, Ozen A, Karakoc-Aydiner E, Chatila TA, Gursel M, Baris S (2021). Stepwise reversal of immune dysregulation due to STAT1 gain-of-function mutation following ruxolitinib bridge therapy and transplantation. J Clin Immunol.

[CR42] Kim SC (2018). Monozygotic twin cases of XIAP deficiency syndrome. J Pediatr Gastroenterol Nutr.

[CR43] Kiykim A, Ogulur I, Dursun E, Charbonnier LM, Nain E, Cekic S, Dogruel D, Karaca NE, Cogurlu MT, Bilir OA, Cansever M, Kapakli H, Baser D, Kasap N, Kutlug S, Altintas DU, Al-Shaibi A, Agrebi N, Kara M (2019). Abatacept as a long-term targeted therapy for LRBA deficiency. J Allergy Clin Immunol Pract.

[CR44] Kostel Bal S, Haskologlu S, Serwas NK, Islamoglu C, Aytekin C, Kendirli T, Kuloglu Z, Yavuz G, Dalgic B, Siklar Z, Kansu A, Ensari A, Boztug K, Dogu F, Ikinciogullari A (2017). Multiple presentations of LRBA deficiency: a single-center experience. J Clin Immunol.

[CR45] Kurolap A, Eshach-Adiv O, Hershkovitz T, Paperna T, Mory A, Oz-Levi D, Zohar Y, Mandel H, Chezar J, Azoulay D, Peleg S, Half EE, Yahalom V, Finkel L, Weissbrod O, Geiger D, Tabib A, Shaoul R, Magen D (2017). Loss of CD55 in eculizumab-responsive protein-losing enteropathy. N Engl J Med.

[CR46] Kurolap A, Eshach-Adiv O, Gonzaga-Jauregui C, Dolnikov K, Mory A, Paperna T, Hershkovitz T, Overton JD, Kaplan M, Glaser F, Zohar Y, Shuldiner AR, Berger G, Baris HN (2018). Establishing the role of PLVAP in protein-losing enteropathy: a homozygous missense variant leads to an attenuated phenotype. J Med Genet.

[CR47] Kurolap A, Eshach Adiv O, Hershkovitz T, Tabib A, Karbian N, Paperna T, Mory A, Vachyan A, Slijper N, Steinberg R, Zohar Y, Mevorach D, Baris Feldman H (2019). Eculizumab is safe and effective as a long-term treatment for protein-losing enteropathy due to cd55 deficiency. J Pediatr Gastroenterol Nutr.

[CR48] Kurolap A, Hagin D, Freund T, Fishman S, Zunz Henig N, Brazowski E, Yeshaya J, Naiman T, Pras E, Ablin JN, Baris Feldman H (2022). CD55-deficiency in Jews of Bukharan descent is caused by the Cromer blood type Dr(a−) variant. Hum Genet.

[CR49] Lee YT, Sung JJY (2004). Protein-losing enteropathy. Gastrointest Endosc.

[CR50] Legendre CM, Licht C, Muus P, Greenbaum LA, Babu S, Bedrosian C, Bingham C, Cohen DJ, Delmas Y, Douglas K, Eitner F, Feldkamp T, Fouque D, Furman RR, Gaber O, Herthelius M, Hourmant M, Karpman D, Lebranchu Y (2013). Terminal complement inhibitor eculizumab in atypical hemolytic-uremic syndrome. N Engl J Med.

[CR51] Lo B, Fritz JM, Su HC, Uzel G, Jordan MB, Lenardo MJ (2016). CHAI and LATAIE: new genetic diseases of CTLA-4 checkpoint insufficiency. Blood.

[CR52] Lopez-Herrera G, Tampella G, Pan-Hammarström Q, Herholz P, Trujillo-Vargas CM, Phadwal K, Simon AK, Moutschen M, Etzioni A, Mory A, Srugo I, Melamed D, Hultenby K, Liu C, Baronio M, Vitali M, Philippet P, Dideberg V, Aghamohammadi A (2012). Deleterious mutations in LRBA are associated with a syndrome of immune deficiency and autoimmunity. Am J Hum Genet.

[CR53] Marsh RA, Rao K, Satwani P, Lehmberg K, Müller I, Li D, Kim MO, Fischer A, Latour S, Sedlacek P, Barlogis V, Hamamoto K, Kanegane H, Milanovich S, Margolis DA, Dimmock D, Casper J, Douglas DN, Amrolia PJ (2013). Allogeneic hematopoietic cell transplantation for XIAP deficiency: an international survey reveals poor outcomes. Blood.

[CR54] Masia R, Peyton S, Lauwers GY, Brown I (2014). Gastrointestinal biopsy findings of autoimmune enteropathy: a review of 25 cases. Am J Surg Pathol.

[CR55] Meesilpavikkai K, Dik WA, Schrijver B, Nagtzaam NMA, Posthumus-van Sluijs SJ, van Hagen PM, Dalm VASH (2018). Baricitinib treatment in a patient with a gain-of-function mutation in signal transducer and activator of transcription 1 (STAT1). J Allergy Clin Immunol.

[CR56] Merico D, Pasternak Y, Zarrei M, Higginbotham EJ, Thiruvahindrapuram B, Scott O, Willett-Pachul J, Grunebaum E, Upton J, Atkinson A, Kim VHD, Aliyev E, Fakhro K, Scherer SW, Roifman CM (2021). Homozygous duplication identified by whole genome sequencing causes LRBA deficiency. NPJ Genom Med.

[CR57] Meyers CD, Amer A, Majumdar T, Chen J (2015). Pharmacokinetics, pharmacodynamics, safety, and tolerability of pradigastat, a novel diacylglycerol acyltransferase 1 inhibitor in overweight or obese, but otherwise healthy human subjects. J Clin Pharmacol.

[CR58] Mudde ACA, Booth C, Marsh RA (2021). Evolution of our understanding of XIAP deficiency. Front Pediatr.

[CR59] Murguia Favela L, Kim VH-D, Upton J, Thorner P, Reid B, Atkinson A, Grunebaum E (2014). IPEX syndrome caused by a novel mutation in FOXP3 gene can be cured by bone marrow transplantation from an unrelated donor after myeloablative conditioning. LymphoSign J.

[CR60] Ostrow AM, Freeze H, Rychik J (2006). Protein-losing enteropathy after fontan operation: investigations into possible pathophysiologic mechanisms. Ann Thorac Surg.

[CR61] Ozen A, Comrie WA, Ardy RC, Domínguez Conde C, Dalgic B, Beser ÖF, Morawski AR, Karakoc-Aydiner E, Tutar E, Baris S, Ozcay F, Serwas NK, Zhang Y, Matthews HF, Pittaluga S, Folio LR, Unlusoy Aksu A, McElwee JJ, Krolo A (2017). CD55 deficiency, early-onset protein-losing enteropathy, and thrombosis. N Engl J Med.

[CR62] Ozen A, Kasap N, Vujkovic-Cvijin I, Apps R, Cheung F, Karakoc-Aydiner E, Akkelle B, Sari S, Tutar E, Ozcay F, Uygun DK, Islek A, Akgun G, Selcuk M, Sezer OB, Zhang Y, Kutluk G, Topal E, Sayar E (2021). Broadly effective metabolic and immune recovery with C5 inhibition in CHAPLE disease. Nat Immunol.

[CR63] Parlato M, Charbit-Henrion F, Elie AN, Begue B, Guegan N, Bruneau J, Khater S, Macintyre E, Picard C, Frédéric RL, le Bourhis L, Allez M, Goulet O, Cellier C, Hermine O, Cerf-Bensussan N, Malamut G (2019). Efficacy of ruxolitinib therapy in a patient with severe enterocolitis associated with a STAT3 gain-of-function mutation. Gastroenterology.

[CR64] Passerini L, Barzaghi F, Curto R, Sartirana C, Barera G, Tucci F, Albarello L, Mariani A, Testoni PA, Bazzigaluppi E, Bosi E, Lampasona V, Neth O, Zama D, Hoenig M, Schulz A, Seidel MG, Rabbone I, Olek S (2020). Treatment with rapamycin can restore regulatory T-cell function in IPEX patients. J Allergy Clin Immunol.

[CR65] Pfundt R, del Rosario M, Vissers LELM, Kwint MP, Janssen IM, de Leeuw N, Yntema HG, Nelen MR, Lugtenberg D, Kamsteeg EJ, Wieskamp N, Stegmann APA, Stevens SJC, Rodenburg RJT, Simons A, Mensenkamp AR, Rinne T, Gilissen C, Scheffer H (2017). Detection of clinically relevant copy-number variants by exome sequencing in a large cohort of genetic disorders. Genet Med.

[CR66] Rantakari P, Auvinen K, Jäppinen N, Kapraali M, Valtonen J, Karikoski M, Gerke H, Iftakhar-E-Khuda I, Keuschnigg J, Umemoto E, Tohya K, Miyasaka M, Elima K, Jalkanen S, Salmi M (2015). The endothelial protein PLVAP in lymphatics controls the entry of lymphocytes and antigens into lymph nodes. Nat Immunol.

[CR67] Rigaud S, Fondanèche MC, Lambert N, Pasquier B, Mateo V, Soulas P, Galicier L, le Deist F, Rieux-Laucat F, Revy P, Fischer A, de Saint Basile G, Latour S (2006). XIAP deficiency in humans causes an X-linked lymphoproliferative syndrome. Nature.

[CR68] Schubert D, Bode C, Kenefeck R, Hou TZ, Wing JB, Kennedy A, Bulashevska A, Petersen BS, Schäffer AA, Grüning BA, Unger S, Frede N, Baumann U, Witte T, Schmidt RE, Dueckers G, Niehues T, Seneviratne S, Kanariou M (2014). Autosomal dominant immune dysregulation syndrome in humans with CTLA4 mutations. Nat Med.

[CR69] Schwab C, Gabrysch A, Olbrich P, Patiño V, Warnatz K, Wolff D, Hoshino A, Kobayashi M, Imai K, Takagi M, Dybedal I, Haddock JA, Sansom DM, Lucena JM, Seidl M, Schmitt-Graeff A, Reiser V, Emmerich F, Frede N (2018). Phenotype, penetrance, and treatment of 133 cytotoxic T-lymphocyte antigen 4–insufficient subjects. J Allergy Clin Immunol.

[CR70] Singhi AD, Goyal A, Davison JM, Regueiro MD, Roche RL, Ranganathan S (2014). Pediatric autoimmune enteropathy: an entity frequently associated with immunodeficiency disorders. Mod Pathol.

[CR71] Smith SJ, Cases S, Jensen DR, Chen HC, Sande E, Tow B, Sanan DA, Raber J, Eckel RH, Farese RV (2000) Obesity resistance and multiple mechanisms of triglyceride synthesis in mice lacking Dgat. http://genetics.nature.com 10.1038/75651 10802663

[CR72] Soler-Palacín P, Garcia-Prat M, Martín-Nalda A, Franco-Jarava C, Rivière JG, Plaja A, Bezdan D, Bosio M, Martínez-Gallo M, Ossowski S, Colobran R (2018). LRBA deficiency in a patient with a novel homozygous mutation due to chromosome 4 segmental uniparental isodisomy. Front Immunol.

[CR73] Speckmann C, Lehmberg K, Albert MH, Damgaard RB, Fritsch M, Gyrd-Hansen M, Rensing-Ehl A, Vraetz T, Grimbacher B, Salzer U, Fuchs I, Ufheil H, Belohradsky BH, Hassan A, Cale CM, Elawad M, Strahm B, Schibli S, Lauten M (2013). X-linked inhibitor of apoptosis (XIAP) deficiency: the spectrum of presenting manifestations beyond hemophagocytic lymphohistiocytosis. Clin Immunol.

[CR74] Stan RV, Tse D, Deharvengt SJ, Smits NC, Xu Y, Luciano MR, McGarry CL, Buitendijk M, Nemani KV, Elgueta R, Kobayashi T, Shipman SL, Moodie KL, Daghlian CP, Ernst PA, Lee HK, Suriawinata AA, Schned AR, Longnecker DS (2012). The diaphragms of fenestrated endothelia: gatekeepers of vascular permeability and blood composition. Dev Cell.

[CR75] Stephen J, Vilboux T, Haberman Y, Pri-Chen H, Pode-Shakked B, Mazaheri S, Marek-Yagel D, Barel O, di Segni A, Eyal E, Hout-Siloni G, Lahad A, Shalem T, Rechavi G, Malicdan MCV, Weiss B, Gahl WA, Anikster Y (2016). Congenital protein losing enteropathy: an inborn error of lipid metabolism due to DGAT1 mutations. Eur J Hum Genet.

[CR76] Strigli A, Gopalakrishnan S, Zeissig Y, Basic M, Wang J, Schwerd T, Doms S, Peuker K, Hartwig J, Harder J, Hönscheid P, Arnold P, Kurth T, Rost F, Petersen BS, Forster M, Franke A, Kelsen JR, Rohlfs M (2021). Deficiency in X-linked inhibitor of apoptosis protein promotes susceptibility to microbial triggers of intestinal inflammation. Sci Immunol.

[CR77] Tesch VK, Abolhassani H, Shadur B, Zobel J, Mareika Y, Sharapova S, Karakoc-Aydiner E, Rivière JG, Garcia-Prat M, Moes N, Haerynck F, Gonzales-Granado LI, Santos Pérez JL, Mukhina A, Shcherbina A, Aghamohammadi A, Hammarström L, Dogu F, Haskologlu S (2020). Long-term outcome of LRBA deficiency in 76 patients after various treatment modalities as evaluated by the immune deficiency and dysregulation activity (IDDA) score. J Allergy Clin Immunol.

[CR78] Thiagarajah JR, Kamin DS, Acra S, Goldsmith JD, Roland JT, Lencer WI, Muise AM, Goldenring JR, Avitzur Y, Martín MG (2018). Advances in evaluation of chronic diarrhea in infants. Gastroenterology.

[CR79] Toubiana J, Okada S, Hiller J, Oleastro M, Gomez ML, Becerra JCA, Ouachée-Chardin M, Fouyssac F, Girisha KM, Etzioni A, van Montfrans J, Camcioglu Y, Kerns LA, Belohradsky B, Blanche S, Bousfiha A, Rodriguez-Gallego C, Meyts I, Kisand K (2016). Heterozygous STAT1 gain-of-function mutations underlie an unexpectedly broad clinical phenotype. Blood.

[CR80] Umar SB, Dibaise JK (2010). Protein-losing enteropathy: case illustrations and clinical review. Am J Gastroenterol.

[CR81] Unsworth DJ, Walker-Smith JA (1985). Autoimmunity in diarrhoeal disease. J Pediatr Gastroenterol Nutr.

[CR82] Uzel G, Sampaio EP, Lawrence MG, Hsu AP, Hackett M, Dorsey MJ, Noel RJ, Verbsky JW, Freeman AF, Janssen E, Bonilla FA, Pechacek J, Chandrasekaran P, Browne SK, Agharahimi A, Gharib AM, Mannurita SC, Joon Yim J, Gambineri E (2013). Dominant gain-of-function STAT1 mutations in FOXP3 wild-type immune dysregulation-polyendocrinopathy-enteropathy-X-linked-like syndrome. J Allergy Clin Immunol.

[CR83] van Rijn JM, Ardy RC, Kuloğlu Z, Härter B, van Haaften-Visser DY, van der Doef HPJ, van Hoesel M, Kansu A, van Vugt AHM, Thian M, Kokke FTM, Krolo A, Başaran MK, Kaya NG, Aksu AÜ, Dalgıç B, Ozcay F, Baris Z, Kain R (2018). Intestinal failure and aberrant lipid metabolism in patients with DGAT1 deficiency. Gastroenterology.

[CR84] Van Wanrooij RLJ, Neefjes-Borst EA, Bontkes HJ, Schreurs MWJ, Langerak AW, Mulder CJJ, Bouma G (2021). Adult-onset autoimmune enteropathy in an European Tertiary Referral Center. Clin Transl Gastroenterol.

[CR85] Villanacci V, Lougaris V, Ravelli A, Buscarini E, Salviato T, Lionetti P, Salemme M, Martelossi S, de Giacomo C, Falchetti D, Pelizzo G, Bassotti G (2019). Clinical manifestations and gastrointestinal pathology in 40 patients with autoimmune enteropathy. Clin Immunol.

[CR86] Vreeburg M, Heitink MV, Damstra RJ, Moog U, van Geel M, van Steensel MAM (2008). Lymphedema–distichiasis syndrome: a distinct type of primary lymphedema caused by mutations in the FOXC2 gene. Int J Dermatol.

[CR87] Wosen JE, Mukhopadhyay D, MacAubas C, Mellins ED (2018). Epithelial MHC class II expression and its role in antigen presentation in the gastrointestinal and respiratory tracts. Front Immunol.

[CR88] Xu L, Gu W, Luo Y, Lou J, Chen J (2020). DGAT1 mutations leading to delayed chronic diarrhoea: a case report. BMC Med Genet.

[CR89] Ye Z, Huang Y, Wang Y, Lu J, Wu J, Yu Z (2019). Phenotype and genotype of a cohort of Chinese children with early-onset protein-losing enteropathy. J Pediatr.

[CR90] Yen CLE, Stone SJ, Koliwad S, Harris C, Farese RV (2008). DGAT enzymes and triacylglycerol biosynthesis. J Lipid Res.

[CR91] Zeissig Y, Petersen B-S, Milutinovic S (2015). XIAP variants in male Crohn’s disease. Gut.

[CR92] Zimmerman O, Rösler B, Zerbe CS, Rosen LB, Hsu AP, Uzel G, Freeman AF, Sampaio EP, Rosenzweig SD, Kuehn HS, Kim T, Brooks KM, Kumar P, Wang X, Netea MG, van de Veerdonk FL, Holland SM (2017). Risks of ruxolitinib in STAT1 gain-of-function-associated severe fungal disease. Open Forum Infect Dis.

